# Perioprosthetic and Implant-Supported Rehabilitation of Complex Cases: Clinical Management and Timing Strategy

**DOI:** 10.1155/2016/2634093

**Published:** 2016-11-07

**Authors:** Luca Landi, Stefano Piccinelli, Roberto Raia, Fabio Marinotti, Paolo Francesco Manicone

**Affiliations:** ^1^Studio di Odontoiatria Ricostruttiva, Rome, Italy; ^2^Dental Laboratory Technician, Studio di Odontoiatria Ricostruttiva, Rome, Italy; ^3^Institute of Clinical Dentistry, Department of Prosthodontics, Catholic University of the Sacred Heart, Rome, Italy

## Abstract

Treatment of complex perioprosthetic cases is one of the clinical challenges of everyday practice. Only a complete and thorough diagnostic setup may allow the clinician to formulate a realistic prognosis to select the abutments to support prosthetic rehabilitation. Clinical, radiographic, or laboratory parameters used separately are useless to correctly assign a reliable prognosis to single teeth except in the case of a clearly hopeless tooth. Therefore, it is crucial to gather the greatest quantity of data to determine the role that every single element can play in the prosthetic rehabilitation of the case. The following report deals with the management of a multidisciplinary periodontally compromised case in which a treatment strategy and chronology were designed to reach clinical predictability while reducing the duration of the therapy.

## 1. Introduction

The treatment planning approach and prosthetic abutment selection have been severely affected by the introduction of end osseous dental implants in daily practice. Implant success has been well documented in fully edentulous [1] and partially edentulous patients [2, 3]. Parallel to the introduction and evolution of osseointegrated implants, all other dental disciplines have evolved both clinically and technologically by significantly raising their level of predictability and success. Therefore, the following question is still to be answered: should we treat periodontally or endodontically compromised teeth or should we extract them and replace them with dental implants?

Despite the efforts of clinicians and researchers, there is still scarce evidence in the literature supporting this choice [4]. Dental implants may be successfully placed in a periodontally compromised patient once periodontitis has been treated and controlled [5, 6], even though this group of patients seems to be more exposed to the risk of developing peri-implantitis compared to a nonperiodontitis group over the long term [6]. Controlling periodontal disease may be achieved only by a strict application of clinical protocols and by placing patients on a strict regimen of maintenance. Due to the chronic nature of the disease, only long-term follow-up may be able to determine the real treatment success [7].

Whenever dental implants are integrated into a complex perioprosthetic rehabilitation, it is critical for the final and long-term success of the therapy to design a treatment strategy in order to monitor the effect of the treatment delivered while shortening the treatment length. The following report deals with the management of a multidisciplinary periodontally compromised case in which a treatment strategy and chronology were designed to reach clinical predictability while reducing the duration of the therapy.

## 2. Case Presentation

A 56-year-old female patient presented in May 2000 in our clinic complaining of functional and esthetic alterations and reporting the progressive migration of several teeth. Her medical history included myocardial infarction about one year earlier. The patient was under pharmacological control for taking antihypertensive, anticlotting, and antiarrhythmic drugs. She used to be a smoker and quit after the heart attack.

## 3. Clinical Exam

Periodontally, the patient presented with clear signs of gingival inflammation with abundant plaque and calculus accumulation in both supra- and subgingival ways and with probing depth ranging from 1 to 10 mm. Teeth migration due to secondary occlusal trauma was evident, and diastema was present between teeth 11 and 12, 11 and 21, and 13 and 14. There was mesial inclination of teeth 11, 15, 17, and 23, and supereruption and buccal inclination of teeth 31, 32, 41, and 42. There was also a faulty restoration (46-*x*-44, 35-*x*-37) and carious lesions on several teeth (13–17).

**Figure 1:**
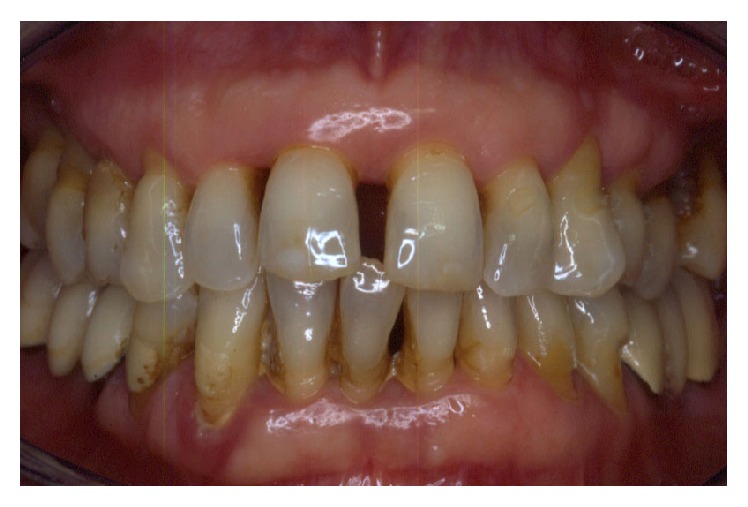
Initial case: clinical frontal view of the patient as she presented in May 2000.

**Figure 2 fig2:**
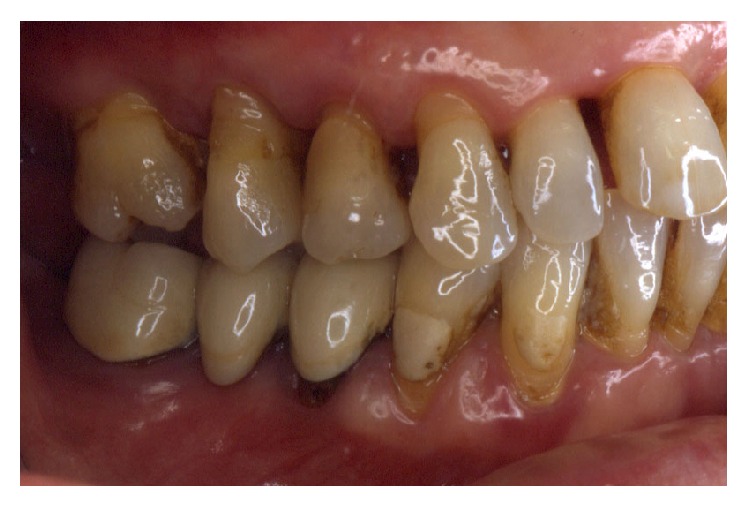
Initial case: lateral view, right side.

**Figure 3:**
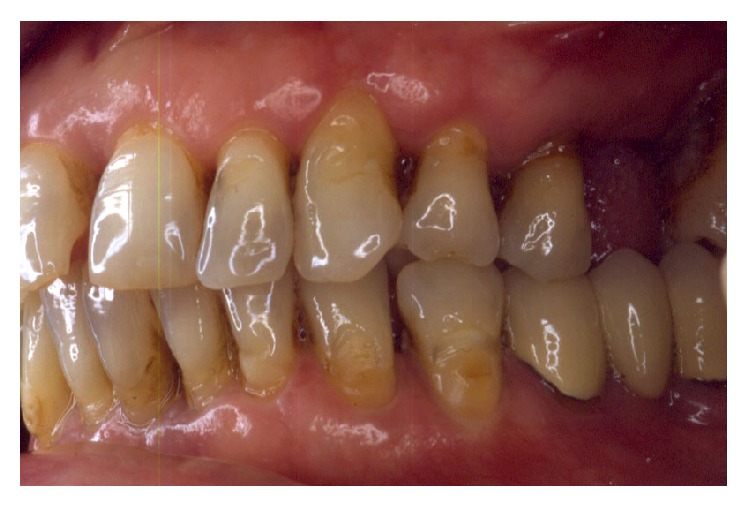
Initial case: lateral view, left side.

The smile line was altered with a buccal inclination of the incisors in relation to the upper lip (Figures 1 [Fig fig2]–3). The clinical frame was therefore characterized by a reduction of function determined by periodontal disease and occlusal instability. Chewing activity was severely compromised and limited by the presence of several mobile and tender teeth.

## 4. Radiographic Examination

**Figure 4:**
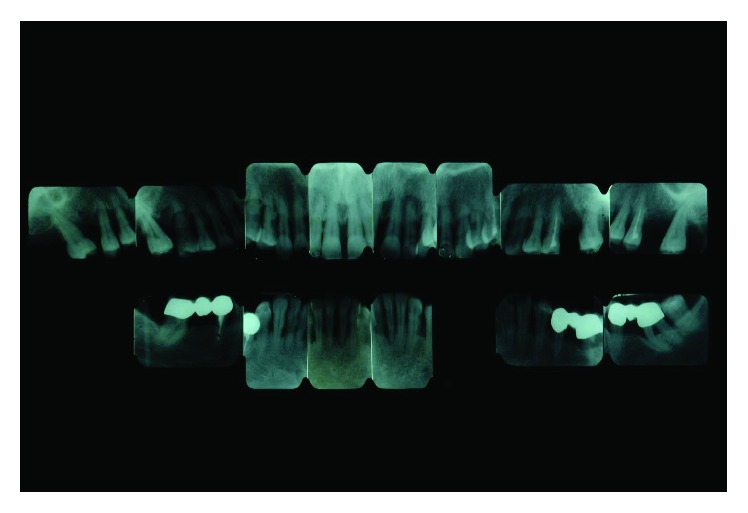
Initial case: full-mouth intraoral radiographic exam (May 2000).

A radiographic loss of about 50% of the alveolar bone was detectable. Teeth 14, 15, 24, and 25 were affected by a circumferential type of defect reaching the apical third of the root. Teeth 17 and 27 had unfavorable root anatomy with an associated vertical alveolar defect leaving only 30–40% of periodontal support. Teeth 21, 34, 35, 43, and 44 presented shallow infrabony defects, and teeth 31 and 41 completely lost the interdental septum (Figure 4).

## 5. Diagnosis

The clinical and radiographic data collected led us to formulate the following diagnosis:

1. Generalized chronic moderate to severe periodontitis (according to the 1999 AAP)
2. Secondary occlusal trauma
3. Caries in teeth 13 and 17
4. Occlusal instability
5. Incompetent lips

## 6. Prognosis

The first step to come up with a complete treatment plan was to formulate a general prognosis and then determine a tooth-by-tooth prognosis in order to select the abutments that could be used for occlusal rehabilitation [8]. From a periodontal standpoint, the general prognosis was good in both the short and long terms.

Due to loss of periodontal support and unfavorable root anatomy, the teeth located in the posterior sextants (17, 27, 37, 38), except tooth 47, had a poor prognosis. The same prognosis was assigned to teeth 14, 15, 24, 25, 31, 32, 41, and 42 for anatomical, periodontal, and endodontic limitations.

## 7. Treatment Goals

Treatment objectives included the reestablishment of periodontal health through the elimination of etiological factors, the creation of a stable occlusal scheme for function, and the enhancement of the esthetic appearance by closing the diastema between teeth 11 and 21 according to the patient's chief complaint.

Periodontal stability may be achieved by bringing probing depth within normal range, reducing inflammatory indices below 10%, as this is a good parameter to prevent disease progression [9] and achieve good plaque control.

In order to maximize patient acceptance and comfort during the treatment, teeth 17 and 27, which were judged hopeless, were scheduled to be used to support a fixed temporary full arch restoration until implant integration would be completed and the case would be ready to be finalized and then extracted. The treatment plan was outlined while taking into consideration the patient's desire to have a normal social and professional life during the treatment and trying to address the patient's chief complaint.

## 8. Phase I

Oral hygiene instructions and motivation combined with several appointments for supra- and subgingival scaling were useful to improve periodontal conditions and test the patient's compliance. Teeth 37 and 38 were extracted due to their hopeless prognosis, whereas the other teeth scheduled for extraction were extracted at the time of temporary restoration. Root canal therapy was done on all the abutment teeth, forecasting an aggressive type of prosthetic preparation at the time of periodontal surgery. A Lucia jig type of appliance was used to occlusally decondition the patient so that, using a bimanual manipulation of the mandible, we were able to accurately detect a centric relation.

Three months after initial consultation and once the patient was considered compliant with the prescribed oral hygiene regimen, the rehabilitation of the case started with the fabrication and delivery of the temporary restorations and the extraction of teeth 14, 15, 24, 25, 31, 32, 41, and 42. Teeth 17 and 27 were used as distal abutments of the cross-arch fixed temporary restoration.

**Figure 5:**
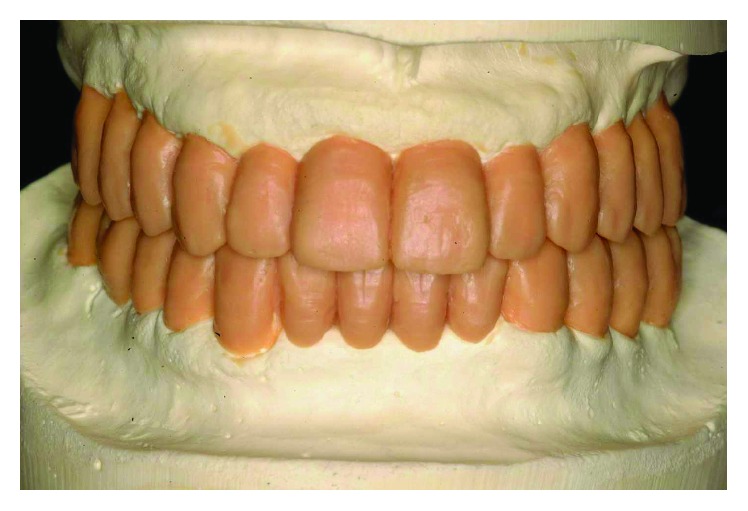
A diagnostic wax-up was made on casts mounted on a semi-individual articulator.

**Figure 6:**
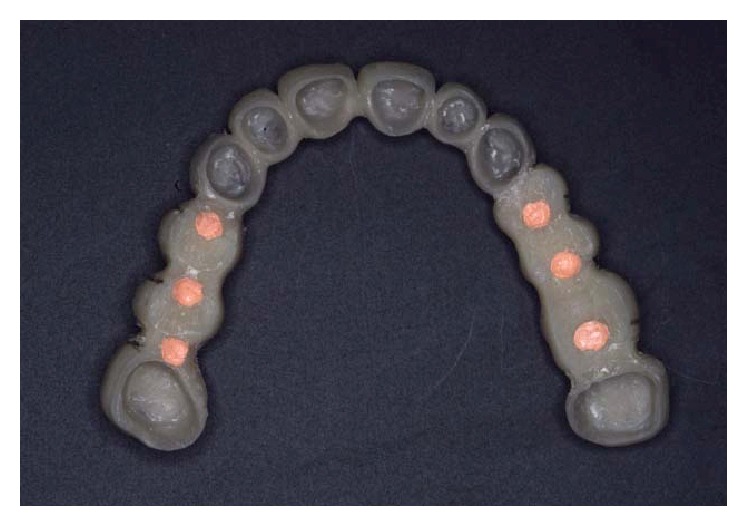
A first set of temporary restorations was developed. The wax-up included implant restorations and radiopaque landmarks embedded in the temporary crowns.

A diagnostic wax-up was made on casts mounted on a semi-individual articulator (Figure 5), and a first set of temporary restorations was developed. The wax-up included implant restorations, and a radiopaque landmark was embedded in the temporary crowns not in relation to the provisional crown but in relation to the final prosthesis (Figure 6). This was done in order to maintain the two maxillary second molars, which were mesially inclined, during the whole treatment. Another advantage of having the radiopaque marking embedded in the temporary crowns was that the patient was able to have a CT scan taken without removing the provisional restorations.

**Figure 7:**
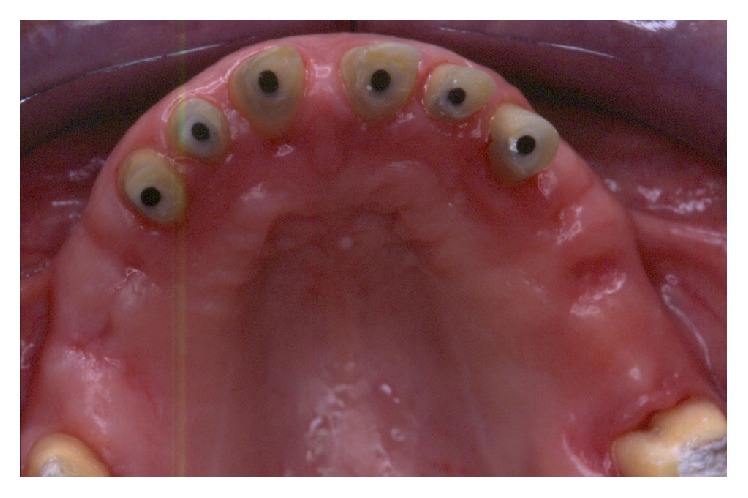
The abutment preparations were done in one appointment with a feather-edge finishing line.

**Figure 8:**
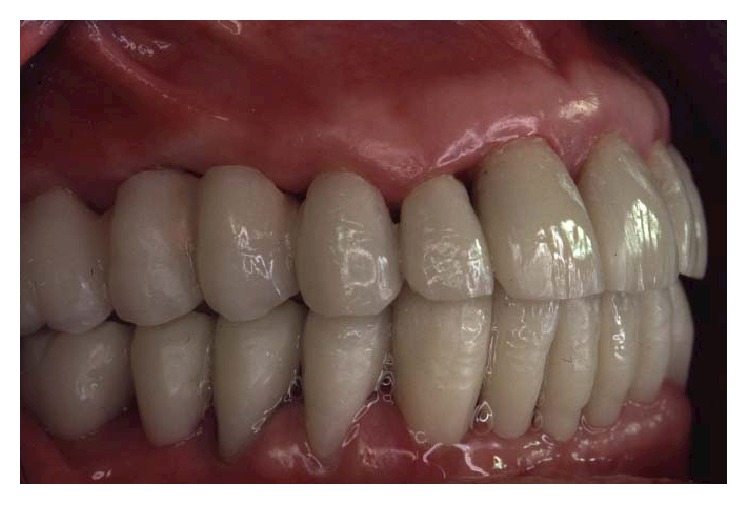
First set of temporary restorations relined and occlusally adjusted.

**Figure 9:**
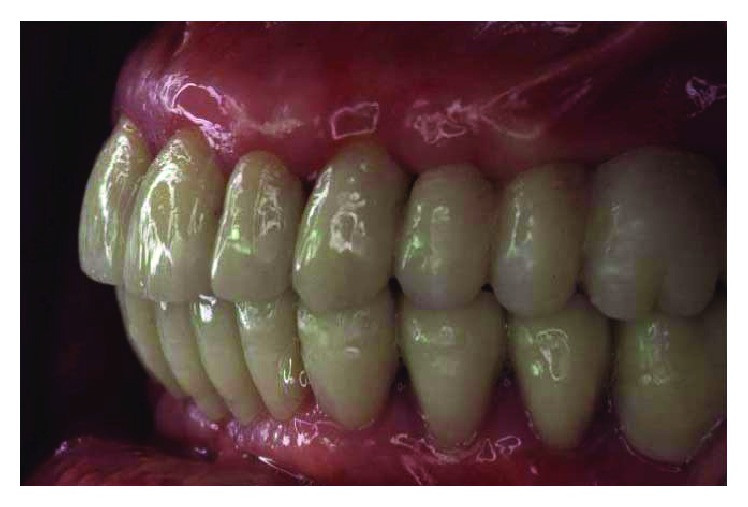
Coincidence between centric occlusion and maximum intercuspation and determination of the incisal plane.

The abutment preparations were done in one appointment with a feather-edge finishing line (Figure 7). The temporary crowns were relined and occlusally adjusted, achieving a coincidence between centric occlusion and maximum intercuspation and carefully controlling the incisal plane (Figures 8 and 9).

## 9. Periodontal Surgery

After nonsurgical periodontal therapy, a periodontal reevaluation was carried out and residual probing depth ranging from 5 to 7 mm was still present particularly in the maxillary anterior sextant. Four months from the beginning of the treatment, osseous resective surgeries were carried out in both the maxillary anterior sextant and the bilaterally posterior mandibular sextants. Main surgical goals were to (1) eliminate periodontal pockets; (2) eliminate infrabony osseous defects; (3) establish a positive bone architecture. In all instances, the intraoperatory abutment preparation according to DiFebo et al. [10] was used in order to achieve better maintenance of the abutments and better tissue healing and to eliminate anatomical root alterations. A brief description of the surgical procedures is reported. Both surgical sessions were conducted under local anesthesia with ultracain supplemented with epinephrine 1 : 100,000 to ensure good hemostasis.

### 9.1. Maxillary Arch

**Figure 10:**
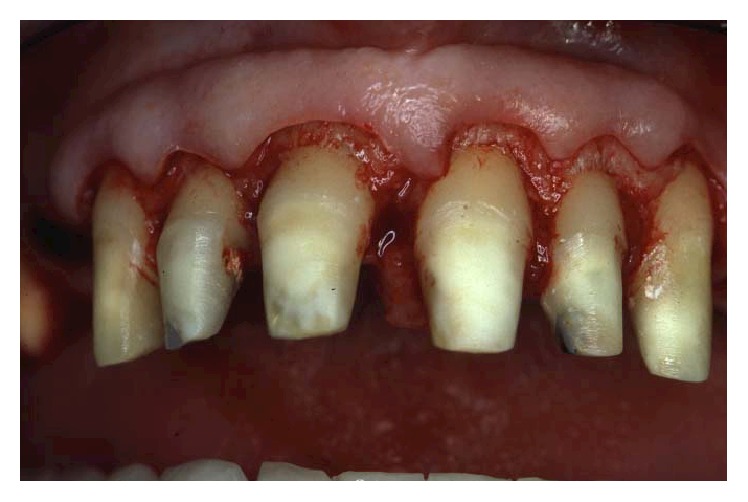
Osseous resective surgery in the maxillary anterior sextant.

**Figure 11:**
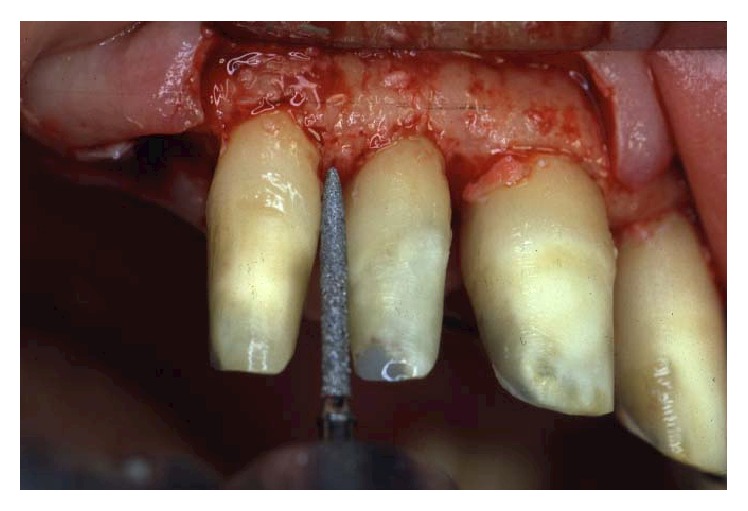
A feather-edge preparation was used for the abutment teeth, deeply modifying the root anatomy and opening the interproximal spaces.

**Figure 12:**
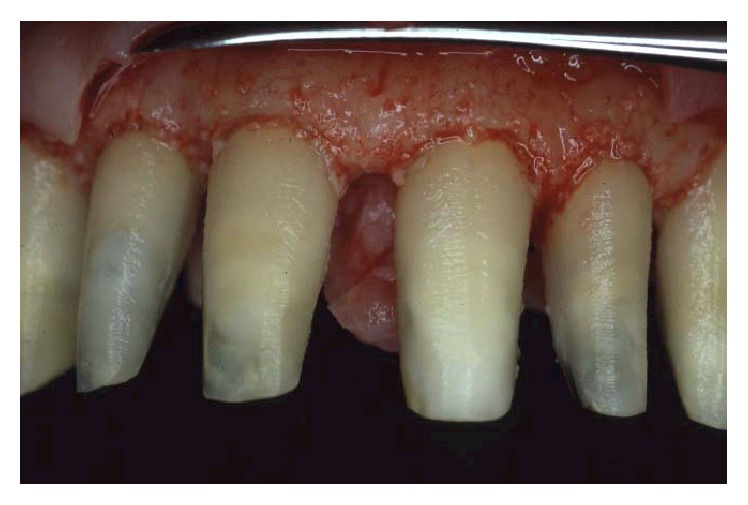
Alveolar bone removal was done until a positive architecture was reached.

**Figure 13:**
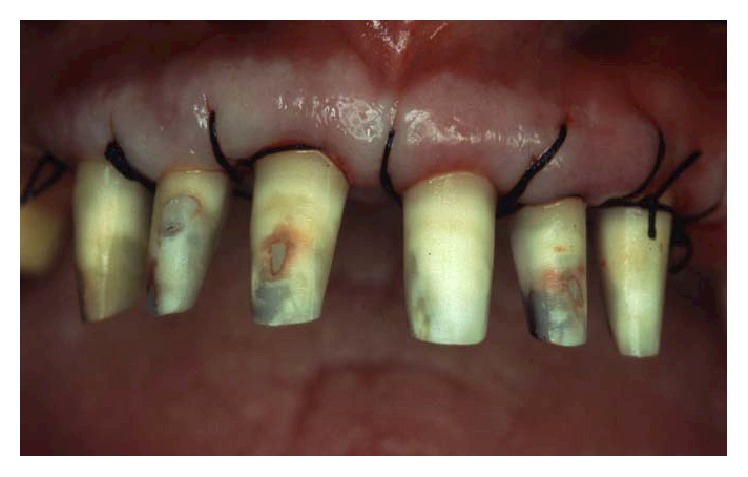
Sling vertical mattress sutures were used to achieve passive flap adaptation to the bone crest.

Buccally, after crestal probing and considering the abundant presence of keratinized gingival, a 2 mm scalloped incision was outlined with a blade number 15 (BD, Bard Parker). A mucoperiosteal flap was raised from teeth 13 to 23 up to the mucogingival line. Palatally, a submarginal beveled incision for crestal anticipation was carried out. The secondary flap and the interproximal tissue were completely removed, and the bone crest was exposed (Figure 10). The deepest part of the intrabony defects was used as a reference to perform osseous resective surgery [11]. Osteoplasty was done using a diamond coarse round bur mounted on a high-speed hand piece under abundant cooling. Ostectomy was achieved mainly using hand-bone chisels. Alveolar bone removal was done until a positive architecture was reached. At this point, a feather-edge preparation was used for the abutment teeth, deeply modifying the root anatomy and opening the interproximal spaces (Figures 11 and 12). Sling vertical mattress sutures were used to achieve passive flap adaptation to the bone crest (Figure 13).

### 9.2. Mandibular Arch

The mandibular left and right sextants were treated simultaneously. Briefly, intrasulcular beveled incisions were outlined, and a split thickness flap was raised in order to preserve the minimal keratinized tissue present in the area. Lingually, a scalloped submarginal full-thickness flap was outlined according to the osseous crest anatomy. Once the interproximal tissue was removed, osseous recontouring was done, and the teeth were prepared as in the maxillary arch. Flaps were moved apically to the bone crest using sling horizontal mattress sutures, trying to increase the amount of keratinized tissue around the abutment teeth.

**Figure 14:**
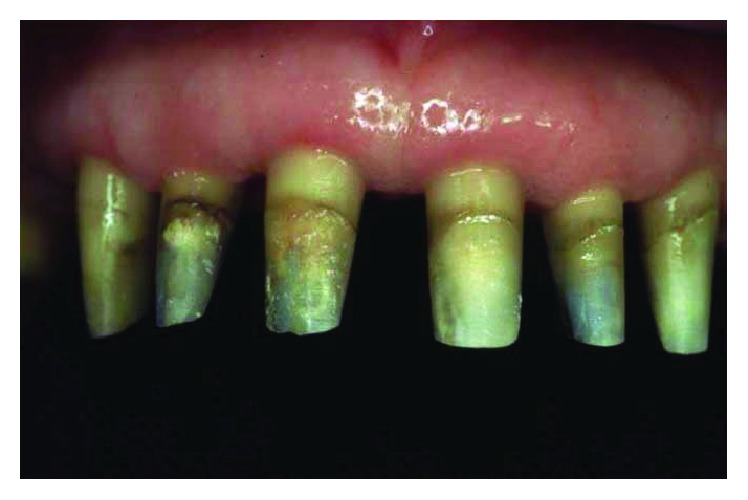
Once the initial healing was completed, 3 months later, the temporaries were relined.

**Figure 15:**
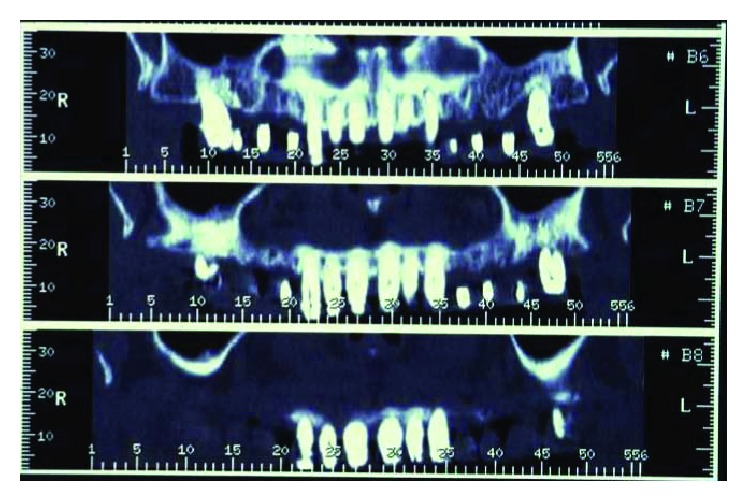
CT scan for evaluation of implant sites.

**Figure 16:**
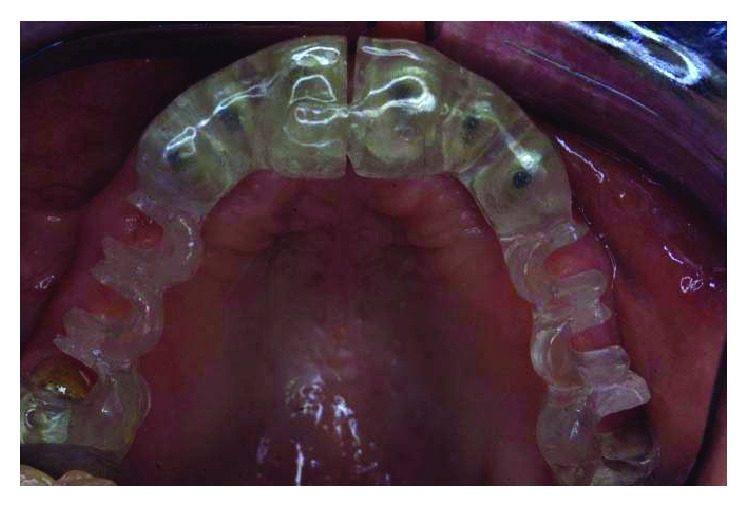
Surgical stents were fabricated based upon the information from the CT scan and using the diagnostic wax-up.

In both cases, temporaries were cemented back at the end of the surgery. The patient was instructed to refrain from brushing the surgical area, to follow a soft diet, and to use a mouthwash (CHX 0.2 twice a day) until mechanical homecare could be resumed. A nonsteroidal anti-inflammatory drug was prescribed (Nimesulide 100 mg,) for the first two days and then only when needed. Healing of the surgical areas was uneventful, with minimal patient discomfort. Once the initial healing was completed, 3 months later (Figure 14), the temporaries were relined, and a new set of precision impressions was taken to fabricate and deliver a second set of temporaries. At this point, the patient was sent for a CT scan for evaluation of the implant sites. Based upon this information and using the diagnostic wax-up, surgical stents were fabricated (Figures 15 and 16).

## 10. Implant Surgery and Temporary Prosthetic Treatment

**Figure 17:**
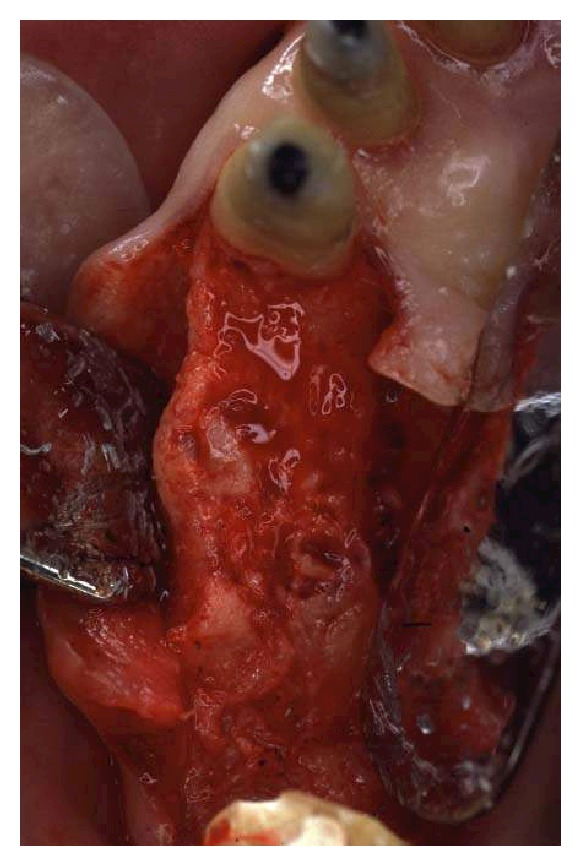
Implant insertion was completed in one surgical session under local anesthesia. Paracrestal full-thickness flaps were elevated.

**Figure 18:**
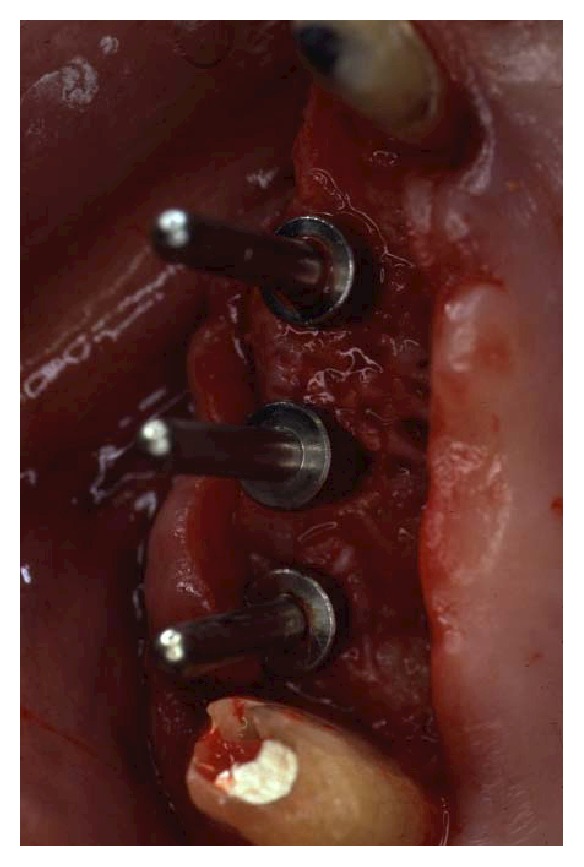
An odontoplasty mesial to tooth 17 was then performed to allocate implant 16 in a prosthetically proper position.

**Figure 19:**
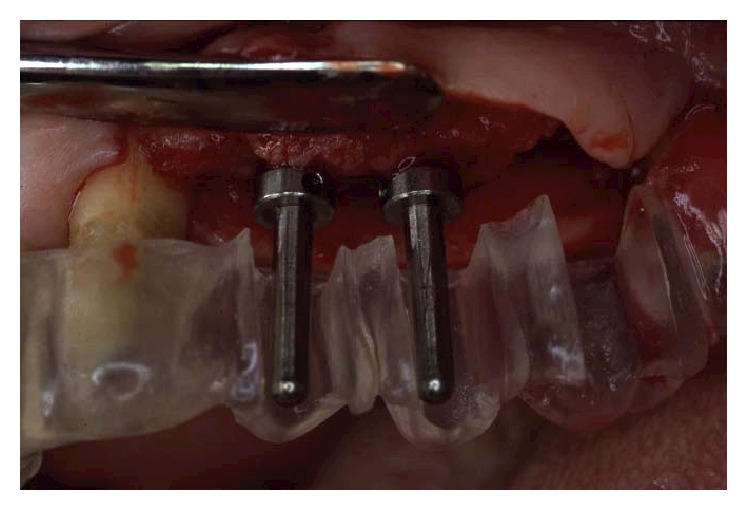
The osteotomy was completed following the surgical stent.

**Figure 20:**
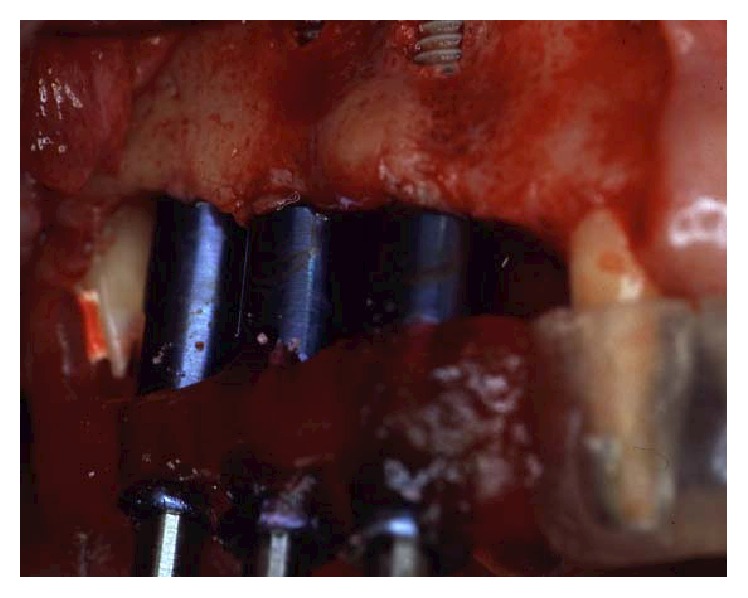
At the time of the insertion, buccal fenestration appeared on implant 14 that required an autologous bone chips graft covered by a resorbable collagen membrane (Biomend).

**Figure 21:**
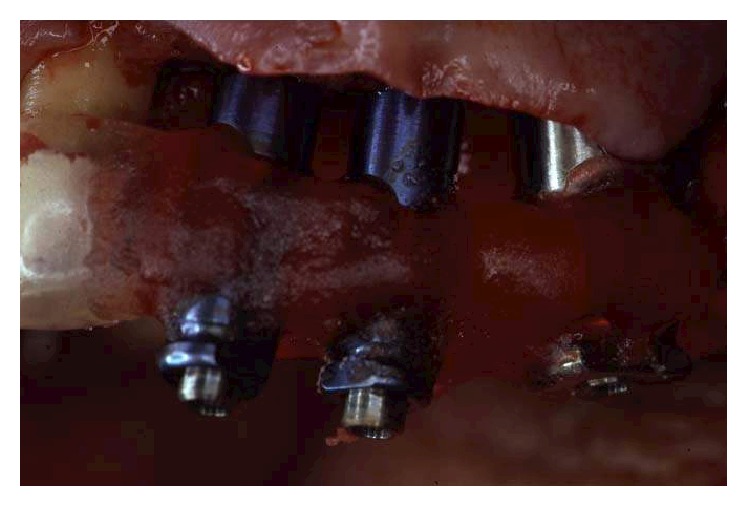
During the surgery, pick-up impression was taken by connecting the implant mounts to the modified surgical stents with a self-polymerizing acrylic resin (Duralay).

One month after periodontal surgical therapy was completed, implant surgery was carried out. Implants insertion was completed in one surgical session under local anesthesia. Paracrestal full-thickness flaps were elevated (Figure 17). Alveolar bone crests were adequate except in two sites where bone defects had to be managed during implant insertion. In implant position 26, there was an adjacent vertical defect mesial to tooth 27. This defect was managed by distally inclining the implant in order to avoid thread exposure distally. The second site was a vertical defect mesial to tooth 17 influencing the insertion of implant 16 (Figure 18). Odontoplasty mesial to tooth 17 was then performed to allocate implant 16 in a prosthetically proper position. Once the osteotomy was completed following the surgical stent (Figure 19), threaded self-tapping 3.75 × 13 mm implants (Twist max Zimmer dental) were screwed into positions 14, 15, 16, 24, 25, 26, and 36. All implants achieved good primary stability with an insertion torque of at least 35 N/cm. At the time of the insertion, buccal fenestration appeared on implant 14 that required an autologous bone chips graft covered by a resorbable collagen membrane (Biomend, Zimmer) (Figure 20). During the surgery, a pick-up impression was taken by connecting the implant mounts to the modified surgical stents with a self-polymerizing acrylic resin (Duralay, Reliance Dental MFG Inc.) (Figure 21).

**Figure 22:**
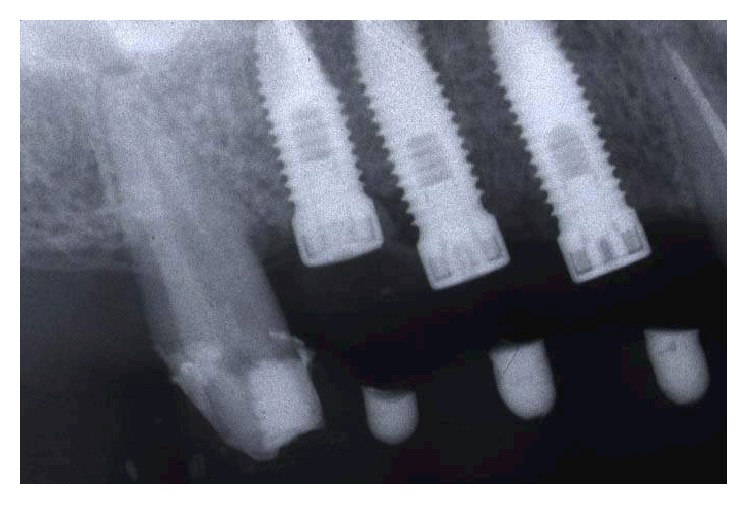
Radiographic postoperative control: coincidence of radiopaque landmarks embedded in the temporary crowns and the implant position of 14, 15, and 16.

**Figure 23:**
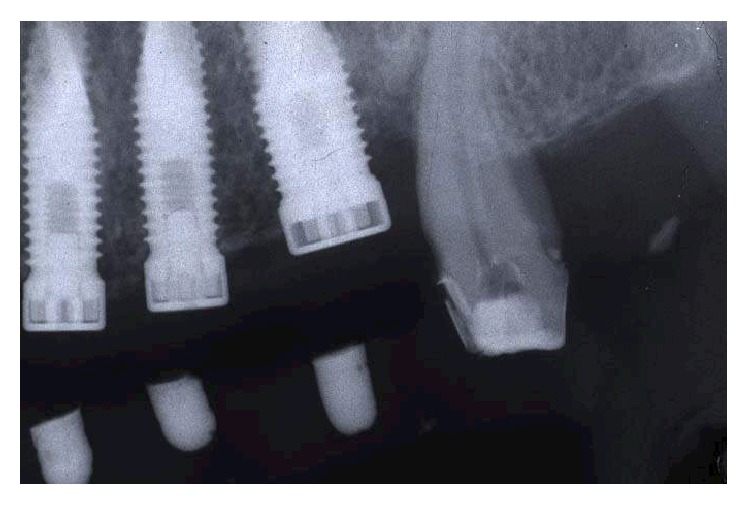
Radiographic postoperative control: coincidence of radiopaque landmarks embedded in the temporary crowns and the implant position of 24, 25, and 26.

**Figure 24:**
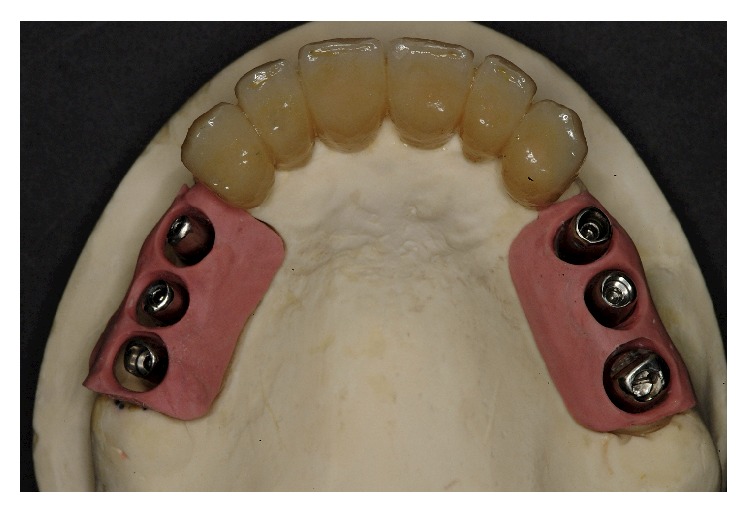
The position of the implants was transferred on the casts used to fabricate the surgical stents.

**Figure 25 fig25:**
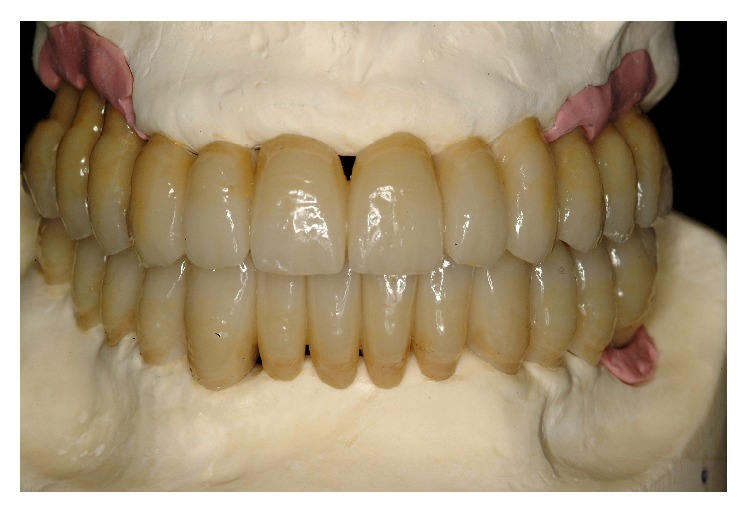
Second set of temporaries: frontal view.

**Figure 26:**
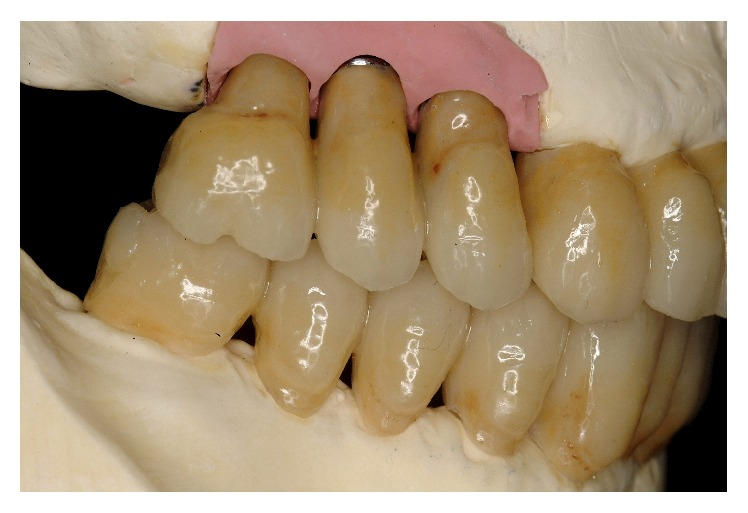
The impression copings used as temporary abutments were milled down according to the prosthetic need, and a second set of temporary restorations was fabricated.

In the radiographic postoperative control, there was a coincidence of radiopaque landmarks embedded in the temporary crowns and the implant positions 14, 15, 16, 24, 25, and 26 (Figures 22 and 23). In the lab, on the casts used to fabricate the surgical stents, the implant position was transferred. The impression copings used as temporary abutments were milled down according to the prosthetic need, and a second set of temporary restorations was fabricated (Figures 24 [Fig fig25]–26).

**Figure 27:**
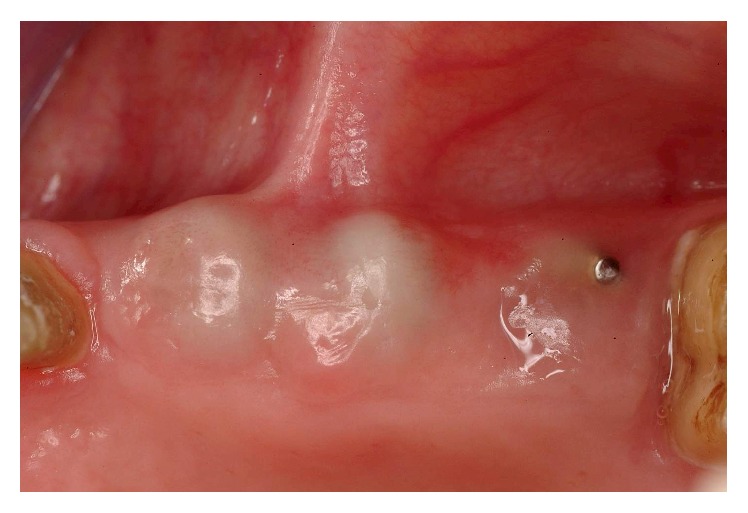
A second stage was performed 5 months after implant surgery.

**Figure 28:**
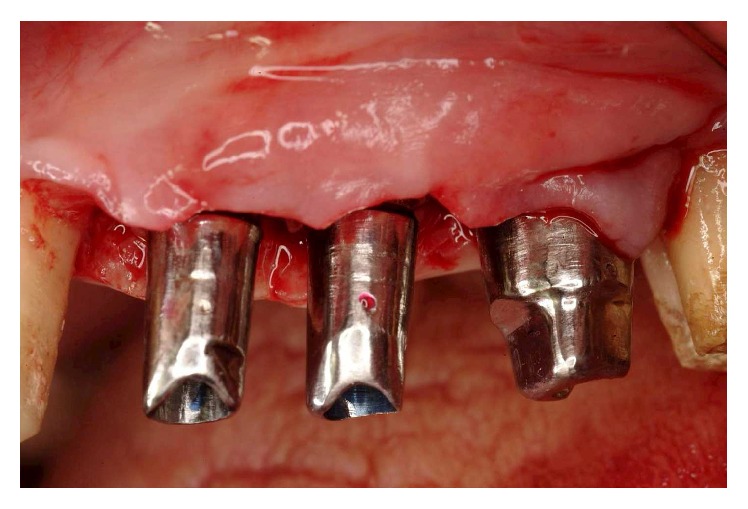
Tightening of the provisional abutments.

**Figure 29 fig29:**
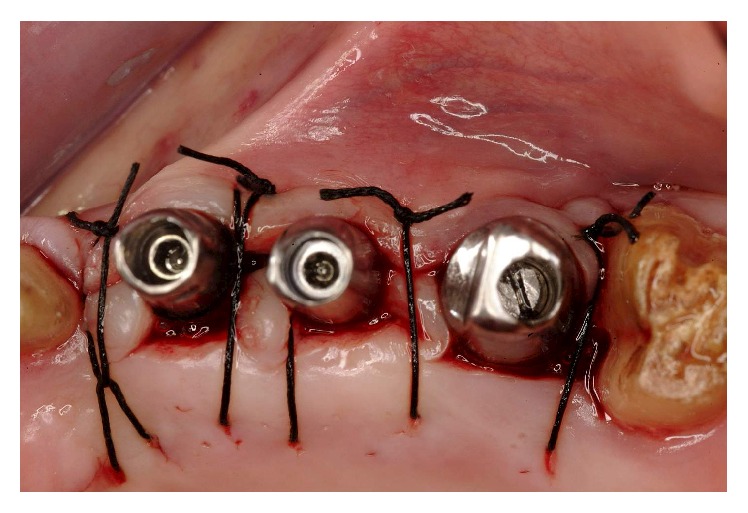
A conservative type of uncovering to preserve and augment the KG tissue present was required.

**Figure 30:**
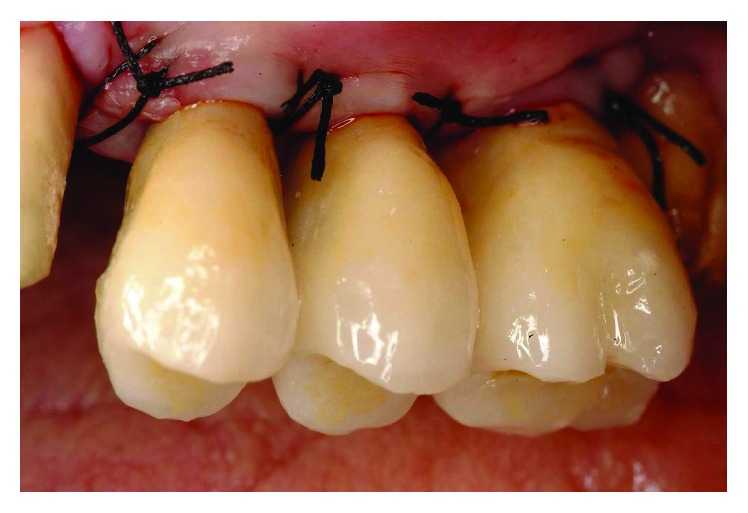
Delivery of the second set of temporaries.

**Figure 31:**
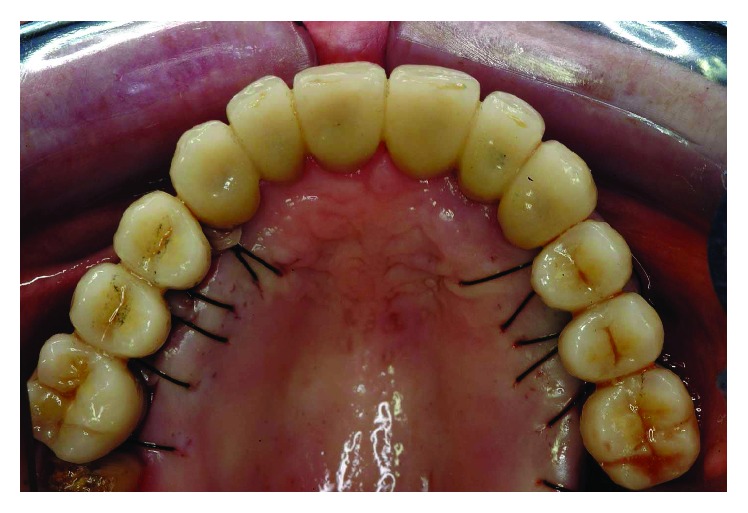
Delivery of the second set of temporaries and extractions of teeth 17 and 27 according to the treatment plan.

Five months after implant surgery, a second stage was conducted. During the healing phase, a spontaneous exposure occurred to some of the implants, requiring a conservative type of uncovering to preserve and augment the KG tissue present (Figure 27). Using the technique described by Palacci [12], the implants were exposed. Provisional abutments were tightened down, and after abutment teeth repreparation the second set of temporaries was delivered (Figures 28 [Fig fig29]–30), thus allowing extractions of teeth 17 and 27 according to the treatment plan (Figure 31).

## 11. Final Restoration

**Figure 32:**
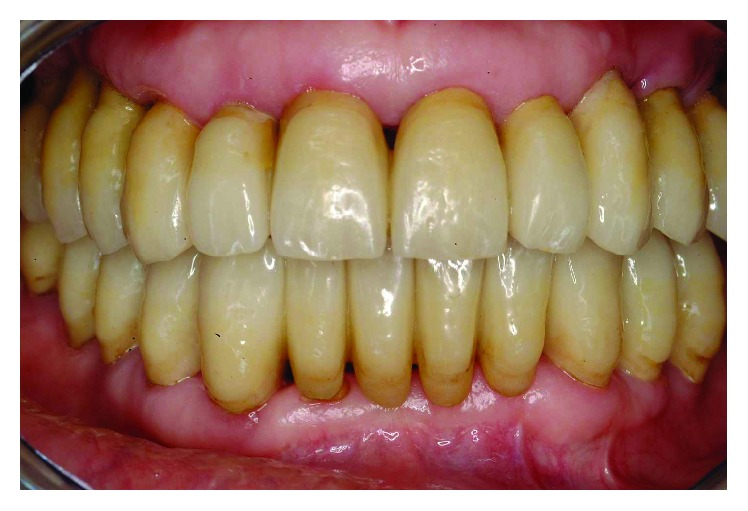
At this point, the patient could be considered stable from a perioprosthetic point of view, waiting 6 more months for a final reevaluation.

**Figure 33:**
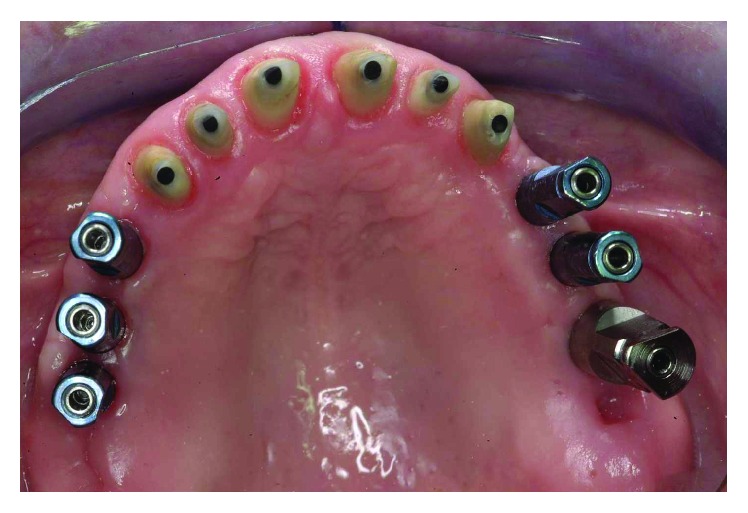
Final preparations from teeth 13 to 23 and positioning of the impression copings for a pick-up impression.

**Figure 34:**
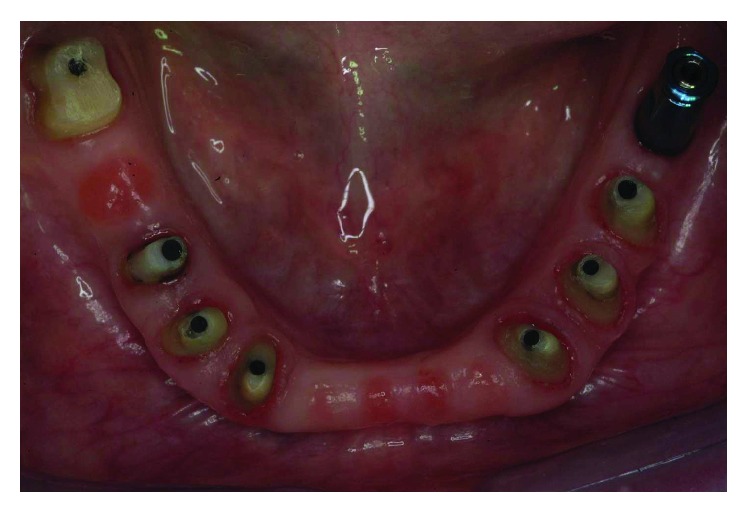
The final impression was performed using a single-phase technique with double components polyether and individual tray.

**Figure 35:**
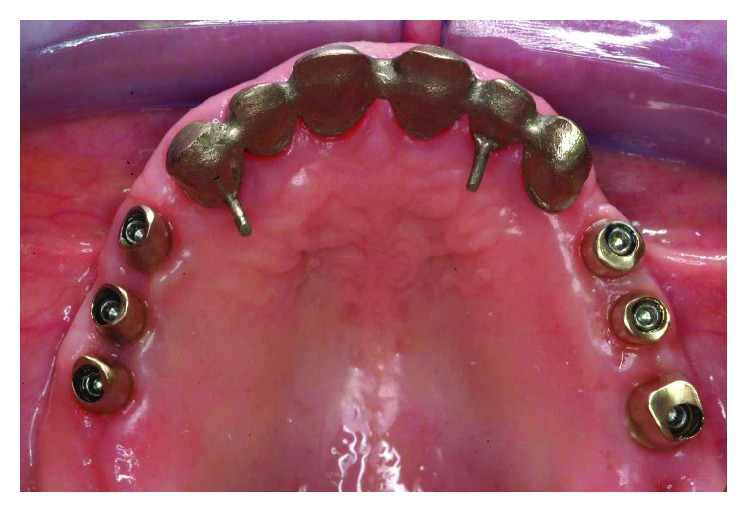
The final restoration included a tooth-borne fixed partial denture from teeth 13 to 23 and two implant-supported fixed partial dentures, one from implant 16 to 14 and the other from implant 24 to 26.

**Figure 36 fig36:**
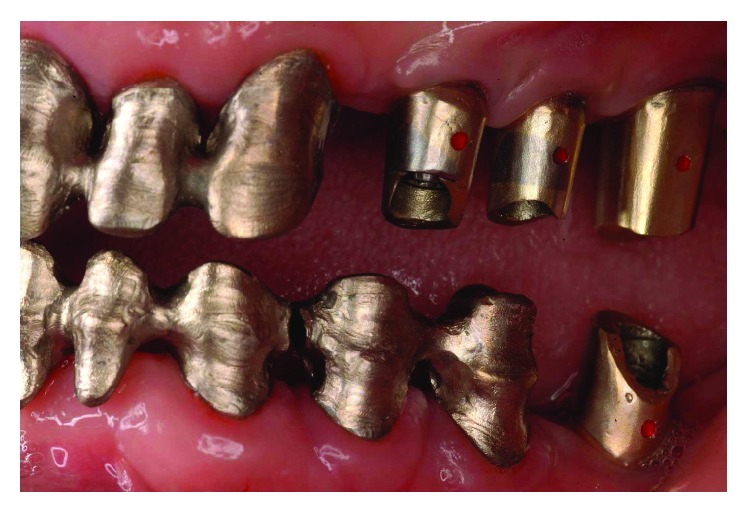
Final framework and ceramization were performed with the cross-mounting technique.

**Figure 37 fig37:**
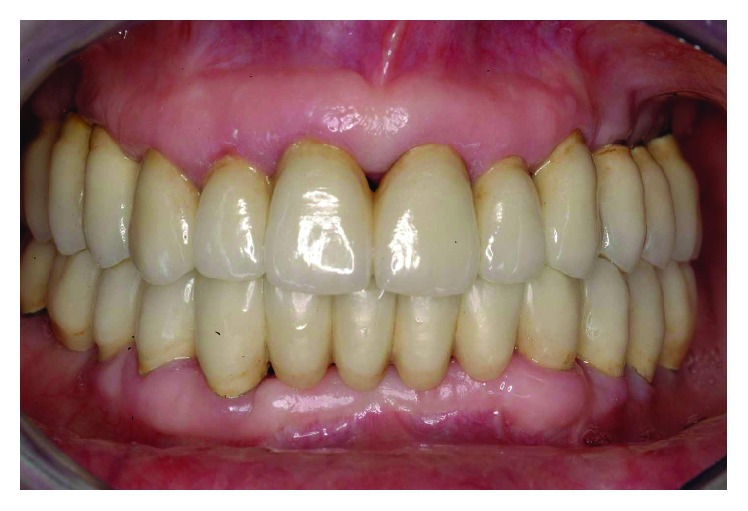
Final delivery of the prosthetic reconstruction (May 2002). From a functional standpoint, the objective of restoring good occlusal stability was achieved.

**Figure 38:**
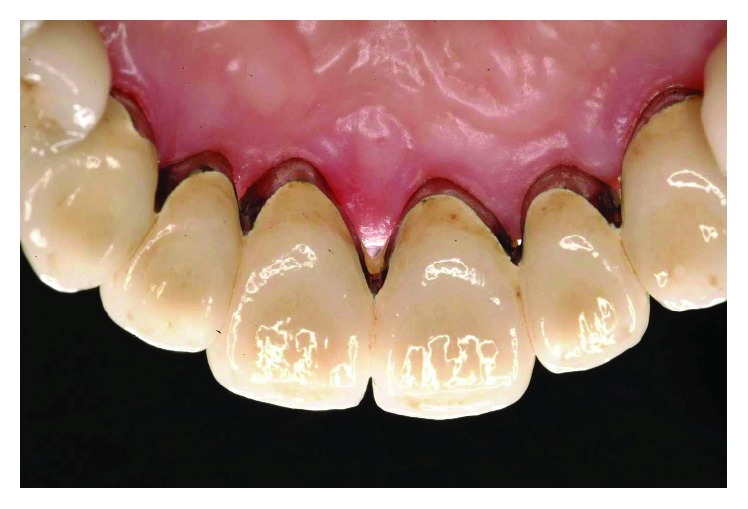
The occlusal scheme was designed with anterior guidance allowing a complete disclusion in both the lateral and protrusive excursions.

At this point, the patient could be considered stable from a perioprosthetic point of view, and we waited 6 more months for a final reevaluation (Figure 32). Periodontal probing was within normal limits, and no inflammation was recorded around the abutment teeth and implants. Thus, a final impression was performed using a single-phase technique with double components polyether and individual tray (Figures 33 and 34). A transferring face-bow with new occlusal registrations was used to mount the casts on a semi-individual articulator. UCLA abutments were used for abutment casting. Final framework and ceramization were performed with the cross-mounting technique: the occlusal scheme was designed with anterior guidance allowing complete disclusion in both the lateral and protrusive excursions. The final restoration included a tooth-borne fixed partial denture from teeth 13 to 23 and two implant-supported fixed partial dentures, one from implant 16 to 14 and the other from implant 24 to 26. In the mandible, a one-piece framework was fabricated splinting teeth 35 to 47, and a single implant supported the PFM crown on 36 (Figures 35 [Fig fig36] [Fig fig37]–38). Prosthetic bridgeworks were delivered two years after initial diagnosis in May 2002.

**Figure 39:**
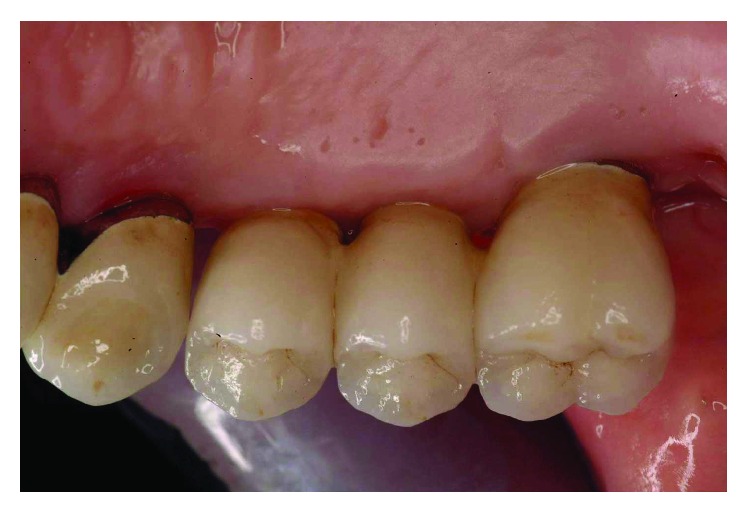
Adequate plaque control, low inflammatory indices, and physiologic probing ranging from 1 to 4 mm around all the abutment teeth and implants were recorded at the end of treatment.

**Figure 40 fig40:**
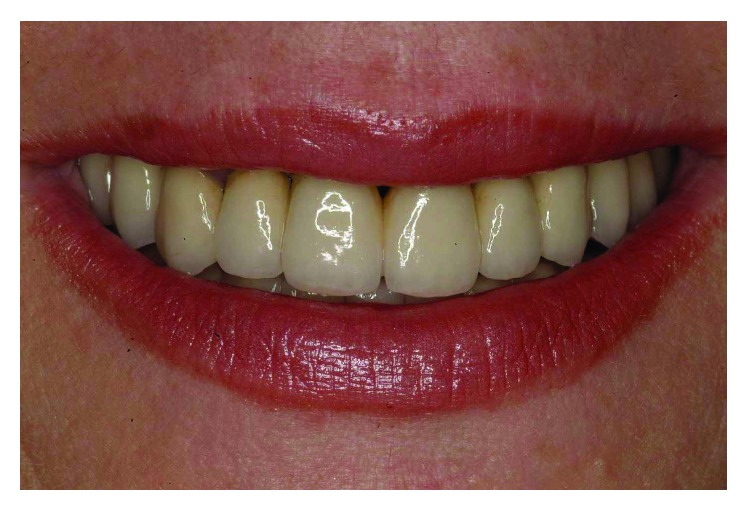
From an esthetic standpoint, the objective of restoring a pleasant and harmonious smile line was achieved.

**Figure 41:**
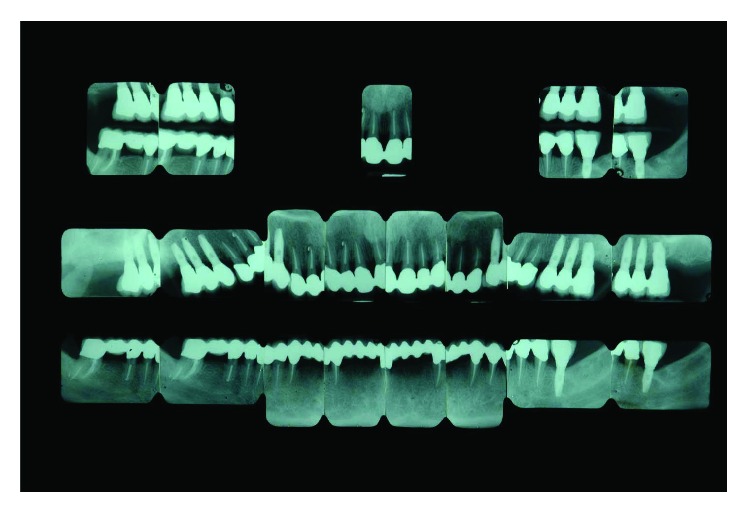
Final case: full-mouth intraoral radiographic exam (May 2002).

At the completion of the treatment, a maxillary retained night guard was provided to prevent any possible negative effect of parafunctional habits. This is in accordance with what has been presented in the literature by Brägger et al. [13] on the incidence of complications in prosthetic success due to parafunctions. From a functional and esthetic standpoint, the objectives were achieved of restoring good occlusal stability and a pleasant and harmonious smile line (Figures 39 [Fig fig40]–41).

The patient was placed on 3 months of supportive periodontal treatment. Periodontal goals were achieved at the end of treatment. Adequate plaque control, low inflammatory indices, and physiologic probing ranging from 1 to 4 mm around all the abutment teeth and implants were recorded at the end of treatment.

## 12. Discussion

Treatment of complex perioprosthetic cases is one of the clinical challenges of everyday practice. Only a complete and thorough diagnostic setup may allow the clinician to formulate a realistic prognosis to select the abutments to support occlusal rehabilitation. According to McGuire and Nunn [8], clinical, radiographic, or laboratory parameters used separately are useless to correctly assign a reliable prognosis to single teeth except in the case of a clearly hopeless tooth. Therefore, it is extremely important to gather the greatest quantity of data, such as probing depth, attachment level, mobility, root anatomy, furcation involvement, inflammatory and hygiene indices, crown-to-root ration, and strategic value, to determine the role that every single element can play in the prosthetic rehabilitation of the case.

The effect of periodontal therapy to preserve compromised and mobile teeth as abutments for complex prosthetic rehabilitation has been widely documented [14]. Those restorations included extensive cantilever in the case of missing molars. Recently, Brägger et al. [13] reported that extensive cantilever should be considered as a true risk factor for failure of fixed partial denture implants or supported teeth. The introduction of oral implants greatly simplified the design of the prosthetic rehabilitation, eliminating the need for a cantilever. However, careful patient selection should be done before implant insertion. Periodontally compromised patients may be eligible for implant therapy only after periodontitis is under control to reduce the risk of developing peri-implantitis [5, 6, 15]. Periodontal control may be achieved by reducing probing within physiologic range, controlling inflammatory indices and plaque scores, and eliminating other potential noxious behaviors such as smoking. In the present case, molars have a negative prognosis due to severe periodontal destruction and poor root anatomy. On the other hand, the anterior maxillary teeth could be treated periodontally. Despite the work of Badersten et al. [16], who were able to manage pockets up to 7 mm in depth with a nonsurgical approach, it is quite clear that, in the case of extensive prosthetic rehabilitation, a more definitive approach should be used. Whenever there is prosthetic commitment in the esthetic zone, the principles of osseous resective surgery may be applied [11, 17]. This treatment modality has been shown to reduce probing for a longer period of time compared to other treatment modalities [18]. The reduction of probing depths has a tremendous impact to shift the periodontal microflora to a nonpathogenic population, thus reducing the risk of peri-implant infection [19].

In the reported case, the use of osseous resective surgery combined with an intrasurgical root preparation [10] resulted in the development of a physiologic hard and soft tissue anatomy compatible with optimal maintenance. Throughout the 24 months of treatment, the patient showed a high level of compliance and good functional and esthetic comfort. Such an extended time frame allowed us to evaluate the patient's response to the treatment and ensure periodontal tissue maturation and stability before the final restorations [20, 21].

The presence of distal teeth allowed us to use those abutments to support conventional temporary cross-arch splinted restorations. Despite the introduction of advanced protocols for implant immediate loading [22] and the use of an enhanced implant surface that may allow healing time reduction [23], we believe it may still be useful to consider hopeless teeth as temporary abutments for extended restorations, provided the teeth can be maintained throughout the treatment free of complications. However, even in a conventional approach, strategies can be implemented to speed up the treatment, such as a pick-up impression of the implant at the time of the surgery. This may also permit the early and progressive functional loading of the implants and the development of a more physiologic soft tissue profile.

## 13. Conclusions

We have reported an advanced periodontally compromised case treated with a multidisciplinary approach. The treatment strategies, rationale, and timing have been presented and explained in detail. Periodontal and prosthetic control of the case and good patient compliance are the key factors for success.
